# Does Bullying Attitude Matter in School Bullying among Adolescent Students: Evidence from 34 OECD Countries

**DOI:** 10.3390/children9070975

**Published:** 2022-06-29

**Authors:** Xiaoou Man, Jiatong Liu, Zengxin Xue

**Affiliations:** School of Humanities and Law, Northeastern University, No. 195 Chuangxin Road, Hunnan District, Shenyang 110169, China; jiatong_liu@outlook.com (J.L.); zengxinxue@outlook.com (Z.X.)

**Keywords:** school bullying, bullying attitude, bullying behavior, adolescent students

## Abstract

There is a need to study the relationship between adolescent bullying attitudes and school bullying behavior to reduce instances of bullying in schools. Based on the Program for International Student Assessment 2018 (PISA 2018), this study investigated the relationship between adolescent bullying attitudes towards different roles and school bullying behavior. Among 34 OECD countries, it also studied the mediating roles of student cooperation and competition, and adolescent bullying attitudes based on gender, grade, and whether one was a bullying victim. We adopted the Coarsened Exact Matching (CEM) method to control the effects of confounders on evaluation results. Overall, the results showed that bullied adolescents’ attitudes towards bullying followers and non-bullied adolescents’ attitudes towards bullying bystanders and defenders were more positively associated with school bullying behavior. Student cooperation partially mediated this relationship and student competition played the suppressor. The findings also provided fresh insights into anti-school bullying campaigns and practices.

## 1. Introduction

School bullying is intentionally aggressive and repetitive behavior targeting individuals with difficulties defending themselves [1,2,3,4]. Although some studies have reported a slight reduction in peer victimization due to the global anti-bullying campaign [5], a recent study has examined the prevalence of school bullying from an international comparative perspective and found that nearly one-third of adolescent students experienced bullying [6]. A total of 23% of students were still exposed to bullying at school at least a few times a month in countries from the Organization for Economic Co-operation and Development (OECD) [7]. Extant research demonstrates that adolescents subjected to bullying suffer worse academic performance [8,9], short- and long-term physical and mental disorders [10,11,12,13,14], and higher rates of suicidal behavior [15,16]. Given the complexity of the issue, understanding some of the factors that lead to school bullying has become the priority of policy makers, educators, parents, and researchers. A significant number of studies have explored these factors at the individual level (such as gender, obesity, and substance addiction) [17,18], family level (such as siblings’ relationship, family socioeconomic status, and parents’ personality) [19], and school climate (such as peer interaction and sense of belonging in school) [20]. Despite many studies investigating factors associated with school bullying, minimal research has revealed the link between attitude and behavior in school bullying [21]. Anti-school bullying, however, as Olweus and Limber stated (2010), should change “attitudes, routines, and behavior in the school environment” [1]. The relationship between bullying attitude and bullying behavior in peer victimization requires advanced research.

Bullying attitudes result from one’s perspective on bullying behavior, and a series of emotional reflections on this issue [22]. Current studies on adolescent bullying attitudes have investigated adolescents’ attitudes towards victims and bullies, and found that most adolescents hold anti-bullying attitudes and have a sense of empathy towards victims [22,23,24,25,26,27]. A study on 170 pupils from Grade 7 to Grade 10 in the U.K. has shown that the mean score for support for victims ranged from 11 to 14, and the mean score for pro-bullying ranged from 7 to 8, indicating that most adolescents expressed a relatively high level of support for victims and anti-bullying attitudes [26]. However, attitudes in support of bullying were also reportedly present [24,28], considering the culture of manliness [24] and the misperception of social norms [23]. From an English primary school sample (*N* = 326), Eslae and Smith (2000) found that although students were inclined to positive anti-bullying attitudes on the whole, 42% of students agreed that “most kids who bully do it for a reason,” and 38% of them agreed “I can understand how some children enjoy bullying” [24]. Andreou et al., (2016) also examined bullying attitudes and victimization by sampling 448 primary school students in central Greece and found that the mean score of “pro-bullying” was 18.18, indicating that most students held pro-bullying attitudes [21].

Numerous studies have looked at the roles played by victims and perpetrators in school bullying. However, there is more to consider when evaluating school bullying and bullying attitudes. Defenders, followers, and bystanders also play a huge role, just as Olweus et al., (2010) illustrated in Bullying Circle Theory [1]. Scholars have also argued that adolescents with different roles to play in school bullying had different reactions [29]. The conventional classification of bullying attitudes while considering only two parties, i.e., bullies and victims, is a simplistic dichotomy. One that has dominated research to an extent where only one study, to our knowledge, had discussed attitudes involving bullies and defenders [30]. Nevertheless, it did not consider adolescents’ attitudes towards followers and bystanders, who also play vital roles. There is, therefore, a need to further analyze the bullying attitudes of all the characters involved.

Existing studies have examined the relationship between attitude and behavior in school bullying according to social psychology theory while considering the effects of adolescent bullying attitudes towards peer victimization [31]. Unsurprisingly, adolescents in support of bullying are more likely to be involved in bullying [21,25,27,32]. Espelage et al., (2017) investigated 310 middle school students in the U.S. and found that those with a pro-bullying attitude were likely to associate with verbal and relational bullying [25]. From a Finnish elementary school student sample (*N* = 1220), Salmivalli et al., (2016) found that a positive attitude towards bullies fueled bullying behaviors [27]. The same results were also obtained from the research conducted by Andreou et al., (2016) [21] and Boulton et al., (2010) [26]. Van Goethem et al., (2010) examined the link between implicit and explicit bullying attitudes and bullying behavior using a sample of 240 fifth- and sixth-grade primary school students in Netherlands. The results showed that explicit bullying attitudes were likely to associate with bullying behavior while implicit attitudes did not provoke bullying behavior [32].

How do attitudes towards different characters influence students in choosing between prosocial and malicious behavior? Social interdependence theory provides two types of interdependence provoking social behavior, namely cooperation and competition [33], which attempt to explain how bullying attitudes influence bullying behavior. Student cooperation is the ability of adolescents to share things, help and support each other [34]. The level of student cooperation is often associated with prosocial or malicious behavior [35]. A study involving a sample of 636 middle school students conducted by Jenkins et al., (2014) showed the positive association between the defenders and adolescents’ cooperation ability [34]. By contrast, student competition involves oppositional interactions. Several empirical studies reported that a competitive school climate provoked increasingly aggressive behavior. The result of one study with a sample of 687 junior high school students in Canada showed that a classroom environment characterized by high competition was associated with increasing bullying behavior [35]. Andreou et al., (2016) demonstrated that adolescents supporting bullying might hold competitive and manipulative attitudes and a competition-perceived environment is likely to foster aggressive behavior [21]. Some scholars were of the idea that both cooperation and competition contributed more to students’ performance than pure cooperation or competition [36]. However, the effects of cooperation and competition on bullying attitudes and behavior require further exploration.

In conclusion, existing studies have analyzed the relationship between bullying attitudes and specific forms of bullying or bullying behavior. However, further study is still required to examine the influence of bullying attitudes towards different roles on bullying behavior and the potential influencing mechanism in peer victimization. This study attempts to fill the research gaps stated above by examining the impact of adolescent bullying attitudes on different roles played in school bullying behavior. It compares such influence between bullied and non-bullied adolescents using a large-scale student sample among 34 OECD countries. It also analyzes the heterogeneity of subgroups for a better understanding of adolescent bullying attitudes towards different characters, as well as the mediating role of cooperation and competition between adolescent bullying attitudes and school bullying behavior. In summary, this study proposes the following hypotheses:

**Hypothesis** **1:** *Adolescent bullying attitudes are significantly associated with school bullying behavior.*

**Hypothesis** **2:** *There are significant differences in adolescent bullying attitudes influenced by whether one is a victim of bullying or not, gender and grade*.

**Hypothesis** **3:** *Student cooperation and competition play mediating roles between adolescent bullying attitudes and school behavior*.

## 2. Materials and Methods

### 2.1. Data and Sample

The data in this research are from the Program for International Student Assessment (PISA) in 2018, when the PISA analyzed adolescent bullying attitudes for the first time, conducted by the Organization for Economic Co-operation and Development (OECD). The OECD is an intergovernmental international economic organization composed of 38 countries, aiming at jointly addressing economic, social welfare, and governance issues brought by globalization. The PISA adopted a two-stage stratified sample technique within schools in the first stage and systematic sampling across grades and gender in the second stage. The PISA’s data collection methods comprised student, teacher, school, and cognition questionnaires. The PISA assessed students who were enrolled in an educational institution at Grade 7 or higher, regardless of the type of educational institution. The PISA specified exclusion criteria including inaccessibility of students’ school, intellectual or physical disability, and a lack of proficiency and material in the test. In most participating countries, more than 80% of the target population were covered. The key variables were mostly from student questionnaires, which used standardized self-reported tests with multiple-choice questions to measure student education development, mental health, performance in campus, family background, etc. The PISA collected data from 294,527 students in 37 OECD countries (no data from Costa Rica). Then, 3 countries (Austria, Korea and Israel) were deleted due to missing samples of key variables, so data from 206,362 adolescent students aged 15~17 years old from 34 OECD countries were compiled as the final evenly distributed samples.

### 2.2. Measurement

#### 2.2.1. Independent Variables: Adolescent Bullying Attitudes

Adolescent bullying attitudes were measured by using students’ self-reported bullying tests in the PISA 2018. According to Olweus et al., (2010), to further classify adolescent bullying attitudes, adolescent bullying attitudes were categorized into “attitude towards bullying followers” (*AF*), “attitude towards bullying bystanders” (*AB*), and “attitude towards bullying defenders” (*AD*). *AF* was assessed under the statement “*It is wrong to join in bullying*” while *AB* was assessed under “*I feel bad seeing other students being bullied*”. *AD* was assessed under “*It irritates me when nobody defends bullied students*” after principal component analysis (KMO = 0.73, *p* < 0.001).

The higher score represented the more positive attitudes towards the corresponding roles played in school bullying. The lower score of *AF* and *AB* and the higher score of *AD* indicated stronger anti-bullying attitudes and vice versa.

#### 2.2.2. Outcome Variable: School Bullying Behavior

The present study adopted an “index of exposure to bullying” in evaluating school bullying behavior among 34 OECD countries. This index of exposure to bullying provided by the PISA was constructed by three types of students’ experience with bullying: “*Other students left me out of things on purpose*”; “*Other students made fun of me*”; “*I was threatened by other students*”. The index was calculated according to the frequency of different forms of school bullying behavior by the OECD, ranging from −1 to 1. Positive values of this index meant that the students were more exposed to bullying than the average student in OECD countries and vice versa [7].

#### 2.2.3. Mediators: Student Cooperation and Competition

“Student cooperation” was assessed under the following statements:

*“Students seem to value cooperation.”*

*“It seems that students are cooperative.”*

*“Students seem to share that cooperation is important.”*

*“Students feel encouraged to cooperate with others.”*

The degree of student-perceived cooperation with peers was measured as follows: 1 = “Not at all true”, 2 = “Slightly true”, 3 = “Very true”, and 4 = “Extremely true”. The average score of the four questions above was calculated and taken to be a representation of “student cooperation”, in which a higher score meant more cooperation, hence positive peer relation. Cronbach’s alpha of student cooperation was 0.91, indicating that the scale had a high reliability.

“Student competition” was assessed under the following statements:

*“Students seem to value competition.”*

*“It seems that students are competing with each other.”*

*“Students seem to share the feeling that competition is important.”*

*“Students feel that they are being compared with others.”*

Students’ perceptions on competition were measured from 1 (“Not at all true”) to 4 (“Extremely true”). The mean score was calculated and taken to represent “student competition”, where a higher score signified a more competitive school climate. Cronbach’s alpha was 0.84 for student competition.

#### 2.2.4. Covariates

Covariates included individual- and family-level characteristics related to school bullying. Individual-level variables included “gender” (0 = Female, 1 = Male), “grade” (0 = Middle school, 1 = High school), and “school absenteeism” (0 = No, 1 = Yes). Family-level variables included “family’s economic status”, “parental education”, and “parental involvement”. “Parental education” was measured by father’s and mother’s education levels. “Parental involvement” was analyzed by using the following statements:

*“My parents support my educational efforts and achievements.”*

*“My parents support me when I am facing difficulties in school.”*

*“My parents encourage me to be confident.”*

The results were measured as follows: “1 = Strongly disagree”, “2 = Disagree”, “3 = Agree”, and “4 = Strongly agree”. The mean score was calculated, with a higher score indicating increased parental involvement. Cronbach’s alpha was 0.89.

### 2.3. Analytic Strategy

This study has adopted the Coarsened Exact Matching (CEM) method to control the effects of cofounders on evaluation of results while analyzing the impact of adolescent bullying attitudes on school bullying behavior, and comparing such effects between bullied and non-bullied adolescents. The Coarsened Exact Matching (CEM) method, raised by Iacus et al., (2008) [37], could enhance comparability by controlling the endogenous problem and reducing the imbalance of covariates between the treatment and control groups.

The CEM is a non-parametric matching method that stems from the monotonic imbalance bounding method. It is more effective and offers more explanation than the existing matching methods, such as Propensity Score Matching (PSM) method. The basic steps of the CEM are as follows: first, sort all the covariates into strata; afterwards, coarsen exact match samples according to the CEM algorithm, while making sure to include in every stratum at least one treated unit (bullied group) and a control unit (non-bullied group), unless pruning it; finally, evaluate the effects of adolescent bullying attitudes on school bullying behavior with successfully matched samples.

Moreover, CEM weights are generated to equalize the sample size of the two comparison groups. The indicator L1 is usually measured before and after matching, to check the multivariate imbalance. L1 ranges from 0 to 1, indicating complete balance or imbalance. Decreasing L1 after matching means successful matching. The detailed analysis technique of the current study is as follows:

Firstly, we summarize adolescent bullying attitudes and school bullying behavior with successfully matched samples. Secondly, we examine differences in adolescent bullying attitudes by gender, grade, and whether one is a victim of bullying. Thirdly, we use the CEM combined with OLS Regression Models to examine the effect of adolescent bullying attitudes on school bullying behavior and compare such effects between bullied and non-bullied students. Lastly, we construct Multivariate Multiple Mediation Models to estimate the mediating effect of student cooperation and competition between adolescent bullying attitudes and school bullying behavior. Compared to conventional mediation models, multivariate mediation models consider similar effects of cooperation and competition and examine the impact of each path and its gross effect. We adopt a Bootstrap test to examine whether there is a partial or full mediating effect. All analyses are performed with Stata 16.0 and incorporate matched weights in matched samples.

## 3. Results

### 3.1. Descriptive Analysis

CEM reports show that the multiple imbalance index before and after matching decreases from 0.261 to 0.123 (presented in Table 1). The successful matching rate is 96.03%, indicating that the bullied and non-bullied groups became more balanced and comparable. After matching, the number of bullied and non-bullied matched samples is 101,600 and 96,563, respectively. Table 1 also presents the summary statistics of samples before and after the CEM method. Among the matched samples, over half of them are female (50.64%). Most students are in high school (67.49%) and have not skipped school in the last two weeks (78.24%).

Adolescent bullying attitudes towards bullying followers (*AF*), bullying bystanders (*AB*), and bullying defenders (*AD*) are 1.608 (SD = 0.810), 1.716 (SD = 0.777), and 3.121 (SD = 0.866), respectively, indicating that most students express a negative attitude towards bullying followers and bystanders, and a positive attitude towards bullying defenders. A total of 89.56% of adolescents have a negative attitude towards bullying followers among 34 OECD countries, accounting for the highest percentage. A total of 88.54% of adolescents are opposed to bystanders, and 83.41% of them support defenders. Over four-fifths of students hold anti-bullying attitudes, while 10~20% of them hold pro-bullying attitudes.

**Figure 1:**
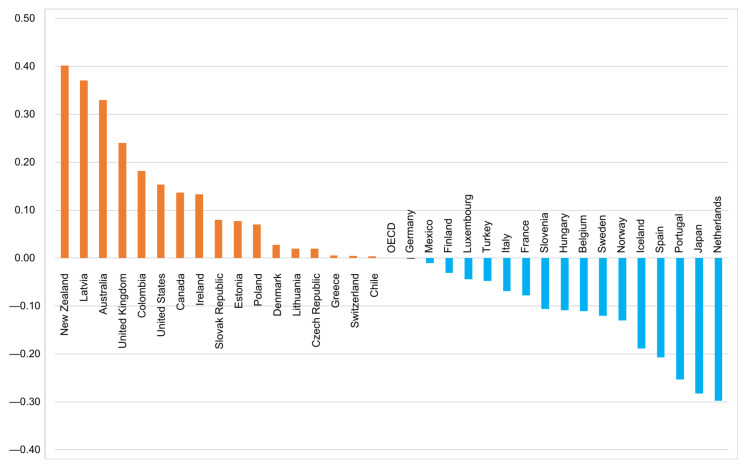
Index of exposure to bullying in 2018 among 34 OECD countries.

Figure 1 shows the index of exposure to bullying among 34 OECD countries in 2018. A positive index of exposure to bullying is found among 17 countries, indicating more frequent school bullying among these 17 countries than at the OECD average level. In contrast, a negative index is found among 17 of the remaining countries.

Table 2 shows adolescent bullying attitudes and basic characteristics of bullied and non-bullied students after the matching process. In terms of adolescent bullying attitudes, students who suffered bullying victimization have lower *AB* (1.706 vs. 1.727, *p* < 0.001) and higher *AD* (3.144 vs. 3.096, *p* < 0.001). *AF* lacks a significant impact on bullied and non-bullied students, partially supporting Hypothesis 2. Students who are male, in middle school, truant, come from a family with higher economic status, lower parental education and lower parental involvement, with lower cooperation and higher competition, are often the bullying target.

### 3.2. Differences among Adolescent Bullying Attitudes, by Gender and Grade

Results of the differences among adolescent bullying attitudes by gender and educational level are presented in Table 3. Female students are more likely to hold negative attitudes towards bullying followers (*AF*) (1.477 vs. 1.741, *p* < 0.001) and bullying bystanders (*AB*) (1.544 vs. 1.893, *p* < 0.001), as well as positive attitudes towards bullying defenders (*AD*) (3.298 vs. 2.939, *p* < 0.001) than male students. In terms of school grade differences, high school students generally express opposition to attitudes supporting bullying followers (*AF*) (1.561 vs.1.703, *p* < 0.001) and bullying bystanders (*AB*) (1.664 vs. 1.823, *p* < 0.001), as well as positive attitudes supporting bullying bystanders (*AD*) (3.164 vs. 3.030, *p* < 0.001) than middle school students, supporting Hypothesis 2.

### 3.3. The Effect of Adolescent Bullying Attitudes on School Bullying Behavior

Table 4 illustrates the effect of adolescent bullying attitudes on school bullying behavior using the matched samples with CEM weights by adopting the Ordinary Least Square Regression method. Results have shown that: (1) attitudes towards bullying followers (*AF*) are likely to associate with school bullying behavior in both bullied (*p* < 0.001, 95% CI: 0.010, 0.014) and non-bullied groups (*p* < 0.001, 95% CI: 0.009, 0.013). The effect of *AF* on school bullying behavior is higher by 0.1% when students have been exposed to bullying. (2) Attitudes towards bullying bystanders (*AB*) are positively associated with school bullying behavior in the non-bullied group (*p* < 0.001, 95% CI: 0.003, 0.007), while *AB* has no significance in the bullied group. (3) Attitudes towards bullying defenders (*AD*) are also positively associated with school bullying behavior in both the bullied (*p* < 0.001, 95% CI: 0.002, 0.005) and non-bullied groups (*p* < 0.001, 95% CI: 0.004, 0.007). Nonetheless, the impact of *AD* on school bullying behavior is lower by 0.2% when students have experienced bullying. In total, attitudes towards bullying followers (*AF*) and bullying defenders (*AD*) are positively associated with school bullying behavior in both the bullied and non-bullied groups, and attitudes towards bullying bystanders (*AB*) are positively associated with school bullying behavior in the non-bullied group. Hypothesis 1 has thus been verified.

Additionally, the influence of student cooperation and competition on school bullying behavior is also examined. When student cooperation increases by 1 unit, school bullying behavior decreases by 0.6% (*p* < 0.001, 95% CI: −0.008, −0.004) and 0.7% (*p* < 0.001, 95% CI: −0.009, −0.006) in the bullied and non-bullied groups, respectively. In both the bullied and non-bullied groups, an increase in student competition by 1 unit shows an increase in school bullying behavior by 3.1%.

### 3.4. Mediating Effects of Student Cooperation and Competition

Results of mediating effects (presented in Table 5) and the Bootstrap test indicate that student cooperation plays the role of a partial mediator between attitudes towards bullying followers (*AF*) and school bullying behavior and between attitudes towards bullying bystanders (*AB*) and school bullying behavior. Positive attitudes towards bullying followers and bystanders result in low student cooperation, thereby aggravating school bullying behavior, partially supporting Hypothesis 3.

**Figure 2:**
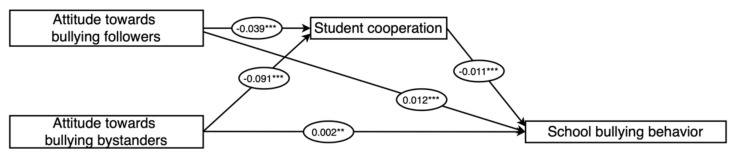
The effect pathways of adolescent bullying attitudes on school bullying behavior. ** *p* > 0.01, *** *p* > 0.001

According to the Bootstrap test, we also find opposite signs of indirect and direct effects on the mediating effects of student cooperation between *AD* and school bullying and mediating effects of student competition between school bullying attitudes and school bullying behavior, indicating that student cooperation plays a suppressor between attitudes towards bullying defenders (*AD*) and school bullying behavior and student competition plays a suppressor between adolescent bullying attitudes and school bullying behavior. The effects on the pathways of adolescent bullying attitudes on school bullying behavior have been shown in Figure 2.

## 4. Discussion

The present study explored the impact of adolescent bullying attitudes on school bullying behavior and the different influences on bullied and non-bullied students. Further, it examined the mediating effects of student cooperation and competition among 34 OECD countries.

Firstly, we found that most adolescents did not support bullying followers (89.56%) and bullying bystanders (88.54%), and were in favor of bullying defenders (83.41%), which indicated that an anti-bullying attitude was the mainstream among students in 34 OECD countries. Only 10~20% of students held a pro-bullying attitude, which was similar to the previous study showing that the percentage of pro-bullying attitude was 20~30% [27]. The difference in the results may have been due to the fact that samples in the current study were adolescents in their high school years who were exposed to school bullying [38]. In addition, we found that the number of countries with frequent school bullying was equal to that with less frequent school bullying among 34 OECD countries relating to social norms, cultural atmosphere and regimes, which deserve further exploration.

Secondly, the results of the heterogeneity analysis showed that: (1) students who have suffered bullying held more negative attitudes towards bullying bystanders and more positive attitudes towards bullying defenders compared to non-bullied students, which is reasonable, given that victimization experiences confirmed negative attitudes towards bystanders and positive attitudes towards defenders. (2) Girls were more likely to express opposition to bullying followers and bystanders and support towards bullying defenders than boys which was consistent with other findings [25,30,39]. In comparison to girls, boys were unconsciously impressed with the “macho stereotype” social norms which were associated with bravery and manliness [24,40]. (3) High school students generally held more negative attitudes towards bullying followers and bystanders and more positive attitudes towards bullying defenders than middle school students in the current study, which was not consistent with some of the findings, leading to the argument that children’s pro-bullying attitude increased with age [26,41]. This inconsistency may be partly due to the difference in students in these samples in terms of age [42], which can easily be explained. Peer norms and class norms tend to have more effects on older adolescents [27]. Adolescent students considered interpersonal networks and class reputation to decide whether to defend victims or support bullies. Some adolescents may adjust their prosocial and anti-bullying attitudes to match with peer group and class general norms facing increasing power from peer pressure. However, when bullying was a tool to punish those threatening class reputation, pro-bullying attitudes may be reasonable and some students may involve in bullying [43,44]. Therefore, it could be effective to build class norms involving peer influences due to the fluid attitude–behavior link.

Thirdly, the current study demonstrated that attitudes towards bullying followers were positively associated with school bullying behavior. This supports the study hypothesis and other findings, which show that followers would reinforce bullying by laughing at victims or perpetrating violence [24,25,26,27,45,46]. Attitudes towards bullying defenders were positively associated with school bullying behavior, a finding that was still open for discussion. Although most students behaved seemingly unconcerned when encountering others victims [27,47], we cannot completely disregard the idea that students holding positive attitudes towards defenders were likely to be victimized by bullies while trying to take action against bullying, thus increasing bullying incidents. A comparison research on the different impacts of adolescent bullying attitudes on school bullying behavior between bullied and non-bullied adolescents was a hint for us to consider that experiencing school bullying may influence adolescent bullying attitudes. Although some researchers have demonstrated that adolescents rarely take action to prevent bullying [26], this conclusion did not consider the impact of bullying victimization on bullying attitudes. Having suffered peer aggression, victims tend to become more against followers. According to social psychology theory, the bullying attitude–behavior link is a complex one due to attitude formation and stability [31,48]. Thus, we continue to face a challenge in anti-bullying intervention and adolescents’ self-defense education and need to explore the bullying attitude–behavior relationship in greater detail.

Last but not least, this study revealed that student cooperation played a mediating role between attitudes towards bullying followers and bystanders and school bullying behavior. The mediating effect analysis also suggested that student competition functioned as a “suppression effect” between adolescent bullying attitudes and school bullying behavior. According to social psychology theory, a person’s behavior is a combination of their internal attitude and the context they confront [49,50]. On the one hand, holding positive attitudes towards followers and bystanders is important, but a cooperative environment and friendly interpersonal relationships are additional factors needed to curb bullying behavior effectively. However, with a competitive and aggressive atmosphere, attitudes cannot change violence, no matter which type of bullying attitudes.

The structure of the PISA 2018, has several limitations that are important to note. The study may have existing sample selection bias and the cross-sectional design of the dataset limits us from establishing a causal mechanism with these variables. A longitudinal design with panel data would be needed for future research to address the link between bullying attitude and bullying behavior in detail. In addition, all variables in the current study are assessed with self-reported data. Although the self-administration questionnaire is one of the main forms of collecting data, future research will try multiple measurement methods from more objective perspectives such as teachers, classmates and parents.

## 5. Conclusions

Despite the above limitations, this study contributed to the existing research on school bullying among adolescents, with considerable strengths. It further categorized bullying attitudes into attitudes towards bullying followers, bystanders and defenders, and compared different effects of adolescent bullying attitudes on school bullying behavior between bullied and non-bullied adolescents with the CEM method. This has not been studied in previous studies. Extant studies mainly focused on the direct effect of bullying attitudes on bullying behavior, whereas our study added to the findings of the mediation mechanism of student cooperation and student competition. The above findings also suggested that further anti-bullying campaigns and practices should target strengthening cooperation, improving adolescent empathy to bullying victims and assisting adolescents to form “belief in a just world” (BJW) from an early age. Furthermore, intervention programs, such as role-play games and cooperative games, help reduce violence and aggression and aid in the development of good attitudes towards different bullying roles played by students. Finally, teach students pro-victim approaches including directly defending victims and indirectly seeking for help.

## Figures and Tables

**Table 1:** Sample summary before and after the Coarsened Exact Matching (CEM) method.

	Before Matching (*N* = 206,362)	After Matching (*N* = 198,163)
	Mean (SD)	Mean (SD)
Attitude towards bullying followers (*AF*)	1.619 (0.819)	1.608 (0.810)
Negative	89.09%	89.56%
Positive	10.91%	10.44%
Attitude towards bullying bystanders (*AB*)	1.726 (0.786)	1.716 (0.777)
Negative	88.09%	88.54%
Positive	11.91%	11.46%
Attitude towards bullying defenders (*AD*)	3.112 (0.872)	3.121 (0.866)
Negative	17.04%	16.59%
Positive	82.96%	83.41%
School bullying behavior	0.009 (0.178)	0.009 (0.178)
Student cooperation	2.645 (0.666)	2.653 (0.665)
Student competition	2.524 (0.672)	2.526 (0.671)
Gender	0.495 (0.500)	0.494 (0.500)
Female	50.53%	50.64%
Male	49.47%	49.36%
Grade	0.674 (0.469)	0.675 (0.468)
Middle school	33.22%	32.51%
High school	66.78%	67.49%
Absenteeism	0.232 (0.424)	0.218 (0.413)
No	76.90%	78.24%
Yes	23.10%	21.76%
Family’s economic status	2.729 (0.479)	2.740 (0.461)
Parental education	1.695 (0.836)	1.658 (0.796)
Parental involvement	3.311 (0.684)	3.339 (0.658)
Multivariate L1	0.261	0.123

**Table 2:** Basic characteristics of bullied and non-bullied groups after matching.

	Treatment Group 95% CI	Control Group 95% CI	*t*-Test/Chi-Square Test
Attitude towards bullying followers (*AF*)	1.606 (1.601, 1.610)	1.610 (1.604, 1.615)	1.097
Attitude towards bullying bystanders (*AB*)	1.706 (1.701, 1.710)	1.727 (1.722, 1.732)	6.083 ***
Attitude towards bullying defenders (*AD*)	3.144 (3.139, 3.150)	3.096 (3.091, 3.102)	−12.377 ***
Student cooperation	2.586 (2.582, 2.590)	2.724 (2.720, 2.728)	46.567 ***
Student competition	2.599 (2.595, 2.603)	2.449 (2.445, 2.454)	−49.873 ***
Gender	0.515 (0.512, 0.518)	0.471 (0.468, 0.475)	−19.210 ***
Grade	0.657 (0.654, 0.660)	0.693 (0.691, 0.696)	17.175 ***
Absenteeism	0.245 (0.242, 0.248)	0.190 (0.187, 0.192)	−29.831 ***
Family’s economic status	2.749 (2.746, 2.752)	2.731 (2.728, 2.733)	−8.752 ***
Parental education	1.643 (1.638, 1.648)	1.673 (1.668, 1.680)	8.296 ***
Parental involvement	3.263 (3.259, 3.267)	3.418 (3.414, 3.422)	52.880 ***

*** *p* < 0.001.

**Table 3:** Differences in adolescent bullying attitudes after matching, by gender and grade.

	Gender		Grade	
Adolescent Bullying Attitudes	Female	Male	Middle School	High School
Attitude towards bullying followers (*AF*)	1.477	1.741	1.703	1.561
*t* test	−73.477 ***		36.654 ***	
Attitude towards bullying bystanders (*AB*)	1.544	1.893	1.823	1.664
*t* test	−1.0 ***		42.686 ***	
Attitude towards bullying defenders (*AD*)	3.298	2.939	3.030	3.164
*t* test	94.377 ***		−32.338 ***	

*** *p* < 0.001.

**Table 4:** Differences in adolescent bullying attitudes after matching, by gender and grade.

	Bullied Group 95% CI	Non-Bullied Group 95% CI
Attitude towards bullying followers (*AF*)	0.012 *** (0.010, 0.014)	0.011 *** (0.009, 0.013)
Attitude towards bullying bystanders (*AB*)	0.002 (−0.000, 0.004)	0.005 *** (0.003, 0.007)
Attitude towards bullying defenders (*AD*)	0.004 *** (0.002, 0.005)	0.006 *** (0.004, 0.007)
Student cooperation	−0.006 *** (−0.008, −0.004)	−0.007 *** (−0.009, −0.006)
Student competition	0.031 *** (0.029, 0.033)	0.031 *** (0.029, 0.032)
Gender	−0.006 ** (−0.009, −0.004)	−0.012 *** (−0.014, −0.010)
Grade	0.0099 *** (0.006, 0.011)	−0.016 *** (−0.019, −0.014)
Absenteeism	0.027 *** (0.025, 0.030)	0.025 *** (0.022, 0.027)
Family’s economic status	0.027 *** (0.024, 0.029)	0.018 *** (0.015, 0.020)
Parental education	−0.016 *** (−0.018, −0.015)	−0.022 *** (−0.024, −0.021)
Parental involvement	0.006 *** (0.004, 0.007)	−0.003 ** (−0.004, −0.001)
Constant	−0.137 *** (−0.150, −0.124)	−0.108 *** (−0.121, −0.095)
R2	0.033	0.038
F	314.49	343.54
Observation	101,600	96,563

** *p* < 0.01; *** *p* < 0.001.

**Table 5:** Mediating effects of student cooperation and competition.

	Outcome Variable	Mediator 1	Mediator 2	Outcome Variable
	School Bullying Behavior	Student Cooperation	Student Competition	School Bullying Behavior
Attitude towards bullying followers (*AF*)	0.013 ***	−0.039 ***	0.022 ***	0.012 ***
Attitude towards bullying bystanders (*AB*)	0.001 *	−0.091 ***	−0.039 ***	0.002 **
Attitude towards bullying defenders (*AD*)	0.007 ***	0.037 ***	0.048 ***	0.006 ***
Student cooperation			0.113 ***	−0.011 ***
Student competition				0.035 ***
R2	0.022	0.082	0.048	0.039
F	493.46	1965.07	994.61	736.33

* *p* < 0.05; ** *p* < 0.01; *** *p* < 0.001.

## Data Availability

The data are available online at https://www.oecd.org/pisa/ (accessed on 10 May 2022).
